# Pharmacokinetics of oral pridinol: Results of a randomized, crossover bioequivalence trial in healthy subjects 

**DOI:** 10.5414/CP203900

**Published:** 2021-04-09

**Authors:** Maren Richter, Frank Donath, Ralph-Steven Wedemeyer, André Warnke, Andreas Horstmann, Claudia Peschel

**Affiliations:** 1 SocraTec R&D GmbH, Oberursel/Erfurt,; 2 SocraMetrics GmbH, Erfurt, and; 3 Strathmann GmbH & Co. KG, Hamburg, Germany

**Keywords:** bioavailability, bioequivalence, immediate-release tablet, pharmacokinetics, pridinol

## Abstract

Objectives: To establish the relative bioavailability and to assess bioequivalence of oral, immediate-release tablets containing pridinol and to determine the pharmacokinetic properties of the compound. Methods and materials: In this single-center, open-label, randomized, crossover trial, healthy male and female adult subjects received single doses of the test and reference product containing 4 mg pridinol mesylate (equivalent to 3 mg pridinol) each under fasting conditions. For pharmacokinetic evaluation, blood samples were withdrawn until 72 hours post dose. Pridinol in plasma was quantified by validated liquid chromatography-mass spectrometry/mass spectrometry (LC-MS/MS). Adverse events (AEs) were analyzed descriptively. Results: Of 34 randomized subjects, 33 completed all treatments. The determined pharmacokinetic parameters were quite similar for both products, with geometric means for maximum exposure (C_max_) of 29.27 ng/mL (test) and 27.44 ng/mL (reference), reached after 1.00 and 0.90 hours (mean t_max_), respectively. The extents of bioavailability (geometric mean AUC_0–tlast_) were 187.93 h×ng/mL (test) and 183.51 h×ng/mL (reference). Elimination half-lives (T_1/2_) ranged from 8.97 to 34.85 hours with comparable mean T_1/2_ of 19.14 hours (test) and 18.85 hours (reference). The point estimates of the test/reference-adjusted geometric mean ratios of AUC_0–tlast_, C_max_ (primary), and AUC_0–∞_ (secondary) were 102.54% (90% confidence interval: 96.19 – 109.32%), 106.79% (99.00 – 115.20%), and 102.60% (96.20 – 109.43%), respectively. Overall, 23 subjects experienced 50 AEs; headache and dizziness (15 cases each) were most frequently reported. Conclusion: Bioequivalence of both pridinol products was demonstrated in terms of rate and extent of absorption. Safety and tolerability were in accordance with the known AE profile of the drug substance.

**What is known about this subject**

- The anticholinergic agent pridinol has been used as a muscle relaxant for decades.
- However, the published literature on the pharmacology of pridinol is sparse.
- More accessible information on the characteristics of pridinol, e.g., its pharmacokinetics in humans, is needed.

**What this study adds**

- This is the first detailed report on the pharmacokinetic properties of pridinol in humans, assisting the prescriber to make informed treatment decisions.
- Bioequivalence of two oral, immediate-release pridinol products was demonstrated.

## Introduction 

Pridinol, a centrally acting muscle relaxant, attenuates polysynaptic reflexes via an anticholinergic mechanism [1, 2]. The compound had been used as a muscle relaxant for decades and is available as a single agent for instance in Germany and Italy. However, the German product (Myoson direct tablets, Strathmann, Hamburg, Germany) was withdrawn from the market in January 2016 due to regulatory reasons. Based on the results of the present study, pridinol-containing tablets were again authorized in Germany in December 2017 (brand name: Myopridin 3 mg tablets, Strathmann, Hamburg, Germany) for treatment of central and peripheral muscle spasms, torticollis, lumbago, and general muscle pain in adults [2]. In 2020, pridinol tablets were approved based on Myopridin as reference in further European countries such as the United Kingdom, Poland, and Spain. 

Skeletal muscle relaxants are a heterogenous drug class used for treatment of central muscle spasms (spasticity), e.g., after stroke, and peripheral musculoskeletal spasms such as those arising from low back pain [1]. In the indication spasticity, pridinol adds to the armamentarium of available muscle relaxants including tizanidine, baclofen, and tolperisone [3, 4, 5]. Of note, in Germany, pridinol is currently – besides methocarbamol [6] – the only approved muscle relaxant for treatment of peripheral muscle spasms associated with low back pain [2], a highly debilitating condition with an estimated prevalence rate between 1.4% and 15.6% [7]. 

The published literature on pridinol is sparse. According to the summary of product characteristics, pridinol reaches its maximum plasma concentration within 1 hour after oral administration and is evenly distributed in tissues [2]. It is metabolized primarily via cytochrome P450 (CYP) 2C19 and CYP2B6 [internal data] to its main metabolite 4-hydroxypridinol [8]. Pridinol is renally eliminated as unchanged drug and as glucuronidated or sulfoconjugated drug [2]. 

The primary objective of the present study was to assess the bioequivalence of two oral pridinol formulations after single-dose administration under fasting conditions. The secondary objectives included the determination of pridinol’s pharmacokinetic characteristics and the assessment of its safety and tolerability. 

## Materials and methods 

### Study participants 

In the study, healthy male or female subjects, aged ≥ 18 years, of Caucasian ethnicity were included. The subjects had a body mass index of 18.5 – 30.0 kg/m^2^ and were non-smokers or ex-smokers for at least 3 months. Pregnant or breast-feeding women were excluded. Furthermore, subjects with contraindications to pridinol and/or conditions that might have an impact on the pharmacokinetics of the compound were excluded. All subjects provided written informed consent before enrollment. 

### Drug products 

Clinical trial batches of the test (Myoson direct, Strathmann, Hamburg, Germany) and the reference product (Lyseen, Novartis Consumer Health, Origgio, Italy) were manufactured according to good manufacturing practice (GMP) standards and were selected in accordance with European requirements for bioequivalence trials [9]. The reference product, registered in Italy, was purchased from the Italian market. 

Both products were divisible, immediate-release tablets containing 4 mg pridinol mesylate corresponding to 3 mg pridinol per tablet as active ingredient. The qualitative composition of the test product included highly dispersed silicon dioxide, hydrogenated castor oil, lactose monohydrate, magnesium stearate, microcrystalline cellulose, polyvinylpyrrolidone, and talcum. Excipients of the reference product were lactose, starch, talcum, and glycerol dibehenate. 

In-vitro dissolution was analyzed according to the Guideline on the Investigation of Bioequivalence [10]. Basket dissolution testing (37 °C, 100 rpm, 500 mL) at pH 1.2, 4.5, and 6.8 revealed a very rapid release with comparable drug release rates of 98.5 – 100.4% (test product) and 98.1 – 99.9% (reference product) after 15 minutes. 

### Study conduct 

The single-center, open-label, randomized (order of treatments), single-dose, crossover trial was conducted from May to July 2016. 

In each period of the trial, the subjects were administered either 1 tablet of the test or reference product in the morning after an overnight fasting period of 8 hours (no food, no beverages, only water was allowed until 1 hour prior to dosing). The use of any systemically available medication except hormonal contraceptives was not allowed. Similarly, specific foods known to interact with metabolizing enzymes (CYP450, P-glycoprotein), e.g., grapefruit/pomelo-containing food or beverages, star fruit-containing food or beverages, St. John’s wort, Brussels sprouts, or broccoli were not permitted. 

Blood samples were collected over 72 hours. This time span was considered adequate to obtain a reliable estimate of the extent of absorption, i.e., the area under the curve (AUC) derived from measurements was expected to cover at least 80% of the AUC extrapolated to infinity (AUC_0–∞_). Since the elimination half-life of pridinol ranged between 3.89 and 24.99 hours in a previous pilot study (Strathmann, Study CPA 139-01, 2002, unpublished), and since individual values of up to 30.5 hours were known from smaller earlier trials (internal data), the washout phase between the 2 treatment periods lasted 13 days to ensure that the drug was virtually completely eliminated from the body prior to subsequent application. 

Testing for bioequivalence (primary objective) was performed considering AUC_0–tlast_ and C_max_. Secondary pharmacokinetic metrics as well as safety and tolerability of the products were investigated descriptively. Standard safety measures comprised vital signs and clinical laboratory parameters assessed prior to inclusion (screening) and at the end-of-trial examination. Adverse events (AEs) within the study (spontaneously reported or upon questioning) were assessed descriptively. 

The clinical trial was conducted in accordance with the Declaration of Helsinki (version 2013), ICH-GCP guidelines (The International Council for Harmonisation of Technical Requirements for Pharmaceuticals for Human Use – Good Clinical Practice), and the requirements of the German Medicinal Products Act (EudraCT no. 2016-001036-35). The trial was approved by the Ethics Committee of the Thuringian Medical Board. 

### Blood samples and preparation 

Blood samples (4.9 mL) for concentration measurement of pridinol were withdrawn within 1.0 hour prior to dosing as well as 0.25, 0.5, 0.75, 1.0, 1.5, 2, 3, 4, 6, 8, 12, 24, 36, 48, 60, and 72 hours post dosing (17 samples per subject and period) and collected in K_2_EDTA tubes. The samples were processed to plasma (centrifugation at 2000 × g, 10 minutes, 4 °C) and subsequently frozen at < –20 °C until analysis. 

### Bioanalytical method validation and sample analysis 

Pridinol in plasma was quantified by liquid chromatography-mass spectrometry/mass spectrometry (LC-MS/MS) after validation according to the Guideline on Bioanalytical Method Validation [9]. Diphenidol hydrochloride served as internal standard. The established lower and upper limits of quantitation for pridinol were 0.0500 ng/mL and 50 ng/mL, respectively. Precision (coefficient of variation (CV): ≤ 4.5%) and accuracy (percentage relative deviation from normal value (RD): ≤ ± 6.6%) during analysis of the trial samples were in accordance with pre-defined acceptance limits [3]. The long-term stability at ≤ –20 °C of 58 days sufficiently covered the longest period for sample storage of 38 days from the first blood sample taken until the last sample analyzed. Likewise, handling of the plasma study samples until measurement was performed within the validated stability time span of 6 hours at room temperature. The incurred sample reanalysis (ISR) passing rate of 87.5% fulfilled the pre-defined criterion of acceptance [3]. 

### Pharmacokinetic and statistical analysis 

Based on internal results from a previous clinical trial comparing 4 mg pridinol mesylate-containing products, intra-individual variabilities of ~ 25% for both AUC and C_max_ were estimated. Setting an α of 5%, considering an apparent ratio of means between test (T) and reference (R) products of µT/µR of 0.95 – 1.05 and acceptance criteria for bioequivalence of 80.00 – 125.00% [10], 28 eligible subjects were needed to achieve a power of 80%. Due to the limited database and to compensate for potential dropouts, 34 subjects were randomized. 

Pharmacokinetic calculations were made using non-compartmental analysis in Phoenix WinNonlin, Version 6.3 (Certara Inc. Princeton, NJ, USA). The primary pharmacokinetic parameters were AUC_0–tlast_ (AUC calculated by the linear-logarithmic trapezoidal method up to the last time point with a quantifiable concentration) and C_max_ (maximum drug concentration measured). Secondary pharmacokinetic parameters included AUC_0–∞_ (AUC extrapolated to infinity), AUC_expol%_ (extrapolated area% calculated as C_last_/λ × 100 / AUC_0–∞_; C_last_ = last quantifiable concentration, λ = apparent terminal elimination rate constant determined by log-linear regression), t_max_ (time from dosing to C_max_), and T_1/2_ (apparent terminal half-life). 

Analyses of variances (ANOVA) were performed as pairwise comparisons of test vs. reference for ln-transformed values of AUC_0–tlast_, AUC_0–∞_, and C_max_ including the factors formulation, period, sequence, and subject(sequence). The relative bioavailability of test vs. reference was assessed by the ratios of geometric means (adjusted, equivalent to the Least Squares Mean) of AUC_0–tlast_, AUC_0–∞_, and C_max_. Bioequivalence was concluded, if the parametric 90% confidence interval (CI) calculated for AUC_0–tlast_ and C_max_ did not exceed the limits of 80.00% and 125.00%. This decision procedure corresponds to two one-sided tests with an error probability α = 0.05 each. Bioequivalence of the two products with respect to AUC_0–∞_ was assessed analogously in a descriptive manner. 

## Results 

### Study participants 

Of 44 enrolled subjects, 34 (17 female and 17 male) subjects, 19 to 55 years of age, were randomized (full-analysis set). The baseline characteristics are depicted in Table 1. One subject dropped out in period II after having completed all planned treatments (reason: withdrawal of consent not related to treatments administered). Thus, this subject remained in the pharmacokinetic evaluation (per-protocol set). Another subject dropped out due to a serious AE not related to the drug product in period I (see safety and tolerability) and was thus excluded from the per-protocol set (N = 33). There were no major protocol deviations such as incorrect inclusion, treatment, or dosing. 

### Pharmacokinetics and bioequivalence 

**Figure 1:**
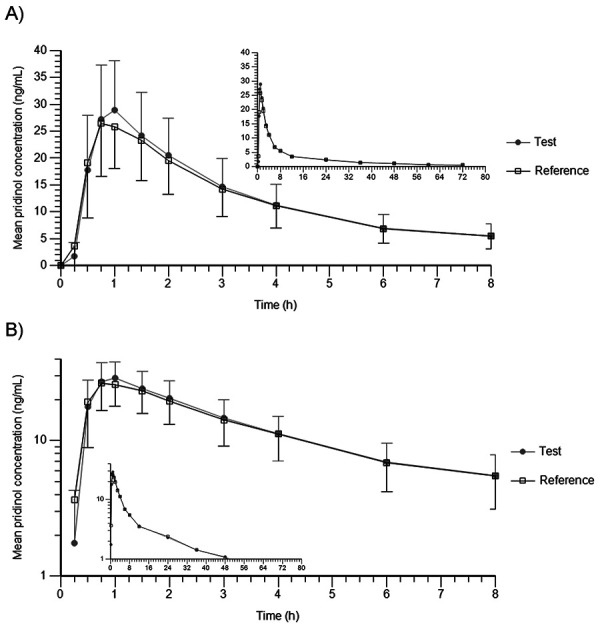
Arithmetic mean plasma concentrations of pridinol after single oral administration of test and reference in 33 healthy subjects (A: linear scale, B: semilogarithmic scale). Shown are means ± standard deviation.

**Figure 2:**
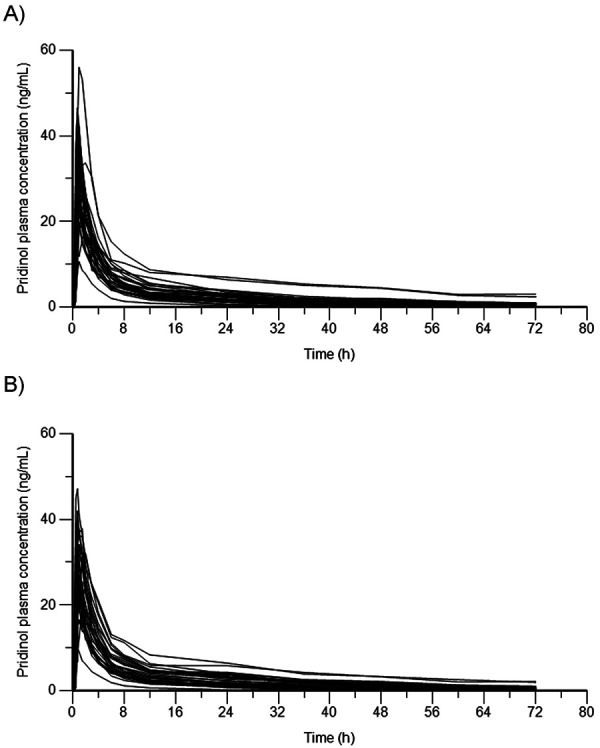
Overlay of individual plasma concentrations of pridinol after single oral administration of A) test (Myoson direct) and B) reference (Lyseen) in 33 healthy subjects.

The mean plasma concentration-time profiles of pridinol after fasted oral administration of test and reference show a highly similar and nearly superimposable course with a steep increase without a lag-time and a maximum (= C_max_) at ~ 1 hour post dosing (Figure 1). The C_max_ is followed by a fast initial decrease until 6 hours post dose to a mean level of ~ 7 ng/mL. Thereafter, the profiles switch to a slower elimination phase until the end of the observed time period of 72 hours to mean values of ~ 0.5 ng/mL. The individual curves (Figure 2) show a quite similar plasma concentration course for all subjects. 

The pharmacokinetic parameters calculated from these profiles are listed in Table 2. The maximum exposure, represented by geometric mean C_max_, was quite similar for both products with 29.27 ng/mL (CV: 33.47%) for test and 27.44 ng/mL (CV: 33.54%) for reference. Likewise, the extent of bioavailability, represented by geometric mean AUC_0–tlast_, was nearly identical for test (187.93 h×ng/mL, CV: 48.16%) and reference (183.51 h×ng/mL, CV: 52.36%). In 1 subject, the extrapolated area (AUC_expol%_) slightly exceeded 20% (22.32% after test, 20.51% after reference). The subject remained in the analysis population since blood sampling over 72 hours is considered sufficient in the sense of a truncated area approach for drugs with a long T_1/2_ [4]. In all other cases, AUC_expol%_ was well below 20%. The mean time points of maximum exposure (t_max_) were comparable for test (1.00 hour post dosing) and reference (0.90 hours post dosing). In all cases, t_max_ was observed after the first sampling time point. The calculated elimination half-lives (T_1/2_) ranged from 8.97 hours to 34.85 hours with comparable mean values for test (19.14 hours) and reference (18.85 hours). 

The point estimates of adjusted geometric means and the affiliated CIs for comparison of test and reference are shown in Table 3. CV_ANOVA_ were low for all analyzed parameters (15.42 – 18.30%). The point estimates for both primary parameters (AUC_0–tlast_ and C_max_) were within the acceptance range of 80.00 – 125.00% suggested by the current guidelines, thus demonstrating bioequivalence between the two products. 

### Safety and tolerability 

No clinically relevant changes in vital signs and laboratory parameters were observed between screening and end-of-study examination. The AEs are summarized in Table 4. After drug product intake, 23 of 34 subjects (67.7%) reported 50 AEs. Of these, 35 AEs (70.0%) were assessed as probably or possibly related to the drug product. The most frequently reported AEs were headache (15 findings in 14 subjects) and dizziness (15 findings in 11 subjects), followed by diarrhea (4 findings in 3 subjects) and nausea (3 findings in 3 subjects). The majority of AEs were of mild (52%) or moderate (40%) intensity. 

One subject reported dizziness of moderate intensity in period I after treatment with the reference drug. Since this AE required inpatient hospitalization for diagnostic measures, it was assessed as serious. However, based on the medical history of the subject, the event was assessed as not related to the drug product. The AE was resolved at the end of the trial. 

Except for 1 AE (subject referred to physician due to increased level of γ-glutamyltransferase), all AEs were resolved at the end of the trial. 

## Discussion 

The present clinical trial was conducted to assess the bioequivalence of the oral immediate-release products Myoson direct (test) and Lyseen (reference), both containing 4 mg pridinol mesylate. The results of this study were the basis for the relaunch of pridinol into the German market in 2017 (brand: Myopridin 3 mg tablets) [2]. 

This is the first detailed report on the pharmacokinetic properties of pridinol in humans. Both pridinol products showed a very similar in-vivo performance as suggested by superimposable plasma concentration profiles and highly comparable pharmacokinetic parameters. All maximum concentrations were recorded well after the first sampling time point. Except for 1 subject, extrapolated areas (AUC_expol%_) did not exceed 20% of AUC_0–∞_ values. In general, blood sampling over 72 hours as done in the present trial is considered sufficient in the sense of a truncated area approach for drugs with a long T_1/2_ [10]. Hence, characterization of the exposure in this trial was in line with the current guideline [10], and the pharmacokinetic results are considered reliable. The bioequivalence of the test and reference product was demonstrated for both primary parameters (AUC_0–tlast_ and C_max_) with point estimates slightly above 100%. The CIs were completely within the conventional acceptance range of 80 – 125%. 

The detected broad range of elimination half-lives (8.97–34.85 hours) may at least be partly explained by intersubject differences in metabolizing enzymes CYP2C19 and CYP2B6 [internal data]. CYP2C19 is subject to genetic polymorphism with a prevalence rate of poor metabolizers ranging between 3% in Europeans and 12 – 22% in Asians [11]. Likewise, pharmacokinetically relevant polymorphisms of CYP2B6 have been described [12]. However, from the therapeutic practice with pridinol for many years, no influence of this inter-subject variability can be deduced on the drug’s safety and efficacy. 

With respect to safety, the type and intensity of AEs observed in this study were in accordance with the safety profile of the drug substance as described in the summary of product characteristics [2]. 

## Conclusion 

This is the first detailed report on the pharmacokinetic characteristics of pridinol. The test product (new brand: Myopridin 3 mg tablets) was found to be bioequivalent to the reference product Lyseen after single-dose, fasting, oral administration. The safety and tolerability of both pridinol preparations were in accordance with the known AE profile of the drug substance. 

## Acknowledgment 

The drug products were provided by the sponsor of the trial (Strathmann GmbH & Co. KG, Hamburg, Germany). The pharmaceutical quality (in-vitro dissolution and content) of the products was checked by Biokirch GmbH, Seevetal, Germany. Bioanalytical analyses were performed by ACC GmbH Analytical Clinical Concepts, Leidersbach, Germany. J. Walstab (SocraTec R&D GmbH, Erfurt, Germany) provided medical writing assistance, supported by Strathmann GmbH & Co. KG. 

## Authors’ contribution 

FD, RSW, AW, AH and CP contributed to conception and design of the study. MR, FD, AD contributed to acquisition of data. MR, FD, RSW, AW contributed to analysis and interpretation of data. All authors revised the article and approved the final version for submission. The authors had complete access to the data that support this publication. They directed and are fully responsible for all content and editorial decisions. 

## Funding 

The clinical trial and the generation of the manuscript were funded by Strathmann GmbH & Co KG, Hamburg, Germany. 

## Conflict of interest 

AW, FD, MR, and RSW were employees of SocraTec R&D GmbH, a CRO acting as contractor of Strathmann GmbH & Co.KG, during conduct of the trial and preparation of the manuscript. AH and CP were employees of Strathmann GmbH & Co.KG during conduct of the trial and preparation of the manuscript. 

**Table 1:** Demographic and anthropometric data of randomized subjects (full-analysis set, N = 34).

	Arithmetic mean (SD)	Median (range)
Age (years)	32 (9)	32 (19 – 55)
Height (m)	1.73 (0.09)	1.75 (1.50 – 1.89)
Weight (kg)	74.1 (11.8)	78.5 (51.3 – 98.4)
BMI (kg/m^2^)	24.5 (2.9)	24.5 (18.7 – 29.9)

BMI = body-mass index; SD = standard deviation.

**Table 2:** Descriptive statistics of pharmacokinetic parameters of pridinol (per-protocol set, N = 33).

	AUC_0–tlast_ (h×ng/mL)		AUC_0–_∞ (h×ng/mL)		AUC_expol%_ (%)		C_max_ (ng/mL)		t_max_ (h)		T_1/2_ (h)	
	Test	Ref	Test	Ref	Test	Ref	Test	Ref	Test	Ref	Test	Ref
Mean (SD)	207.71 (100.42)	204.51 (93.72)	226.72 (128.78)	221.83 (112.90)	6.07 (5.08)	6.04 (4.67)	30.71 (9.34)	28.76 (8.36)	1.00 (0.36)	0.90 (0.27)	19.14 (5.97)	18.85 (5.45)
Median (range)	189.98 (49.68 – 530.28)	182.92 (42.45 – 449.19)	197.11 (50.77 – 682.66)	191.77 (43.20 – 541.91)	5.04 (0.91 – 22.32)	5.04 (0.84 – 20.51)	31.04 (10.64 – 55.94)	27.45 (10.09 – 47.10)	1.00 (0.50 – 2.00)	0.77 (0.52 – 2.02)	18.00 (9.37 – 34.58)	18.21 (8.97 – 32.61)
Geom. mean (CV%)	187.93 (48.16)	183.51 (52.36)	200.38 (52.93)	195.54 (56.48)	4.39 (100.69)	4.47 (98.21)	29.27 (33.47)	27.44 (33.54)	0.95 (31.39)	0.87 (24.61)	18.30 (31.13)	18.11 (29.44)

AUC_0–tlast_ = area under the curve from first to last assessment; AUC_0–_∞ = AUC extrapolated to infinity; AUC_expol%_ = extrapolated AUC%; C_max_ = maximum plasma concentration; CV = coefficient of variation; SD = standard deviation; t_max_ = time from dosing to C_max_; T_1/2_ = apparent terminal half-life.

**Table 3:** Point estimates and confidence intervals of the ratio of adjusted geometric means of the primary and a selected secondary pharmacokinetic parameter (per-protocol set, N = 33).

		90% confidence interval		
Parameter	Point estimate (%)	Lower limit (%)	Upper limit (%)	CV_ANOVA_ (%)
AUC_0–tlast_	102.54	96.19	109.32	15.42
C_max_	106.79	99.00	115.20	18.30
AUC_0–_∞	102.60	96.20	109.43	15.52

ANOVA = analysis of variance; AUC_0–tlast_ = area under the curve from first to last assessment; AUC_0–_∞ = AUC extrapolated to infinity; C_max_ = maximum plasma concentration; CV = coefficient of variation.

**Table 4:** Adverse events after intake of drug products (full-analysis set, N = 34).

N (n)	Test	Reference
AE	13 (21)	20 (29)
Related AE^1^	14 (17)	14 (18)
Serious AE	0	1 (1)
Mild AE	10 (12)	12 (14)
Moderate AE	5 (8)	12 (12)
Severe AE	1 (1)	3 (3)
AEs by SOC		
Nervous system disorders	12 (15)	16 (16)
Gastrointestinal disorders	1 (2)	5 (6)
General disorders and administration site conditions	2 (2)	1 (2)
Injury, poisoning and procedural complications	0	2 (3)
Ear and labyrinth disorders	0	1 (1)
Infections and infestations	1 (1)	0
Investigations	0	1 (1)
Musculoskeletal and connective tissue disorders	1 (1)	0

^1^Possibly or probably related to drug product. AE = adverse event; N = number of subjects; n = number of events; SOC = system organ class.
